# Bind and Separate: How Ephrins and Their Receptors Create Tissue Boundaries

**DOI:** 10.1371/journal.pbio.1001956

**Published:** 2014-09-23

**Authors:** Richard Robinson

**Affiliations:** Freelance Science Writer, Sherborn, Massachusetts, United States of America

At every stage of development, newly formed clusters of cells must separate from others, forming distinct boundaries between them. Without such divisions, there could be no tissue layers, no organs, no bulging muscle contracting against rigid bone.

Accumulating evidence indicates that, in broad outline, the trigger for these types of dissociation is interaction between surface proteins, called ephrins, on one group of cells and ephrin receptors on the opposing group of cells. Binding of the two leads to cell–cell repulsion and eventual separation into distinct tissues. However, cells express ephrins and receptors not only at the latent tissue boundary but throughout the tissue. Given that, it has been unclear how the repulsive signal is restricted to the tissue boundary. In this issue of *PLOS Biology*, Nazanin Rohani, Francois Fagotto, and colleagues elucidate the critical features of ephrin–receptor interaction at the interface between tissues and show that the concentration and binding affinities of specific ligand–receptor pairs largely explain the specificity of this key developmental program.

**Figure 1:**
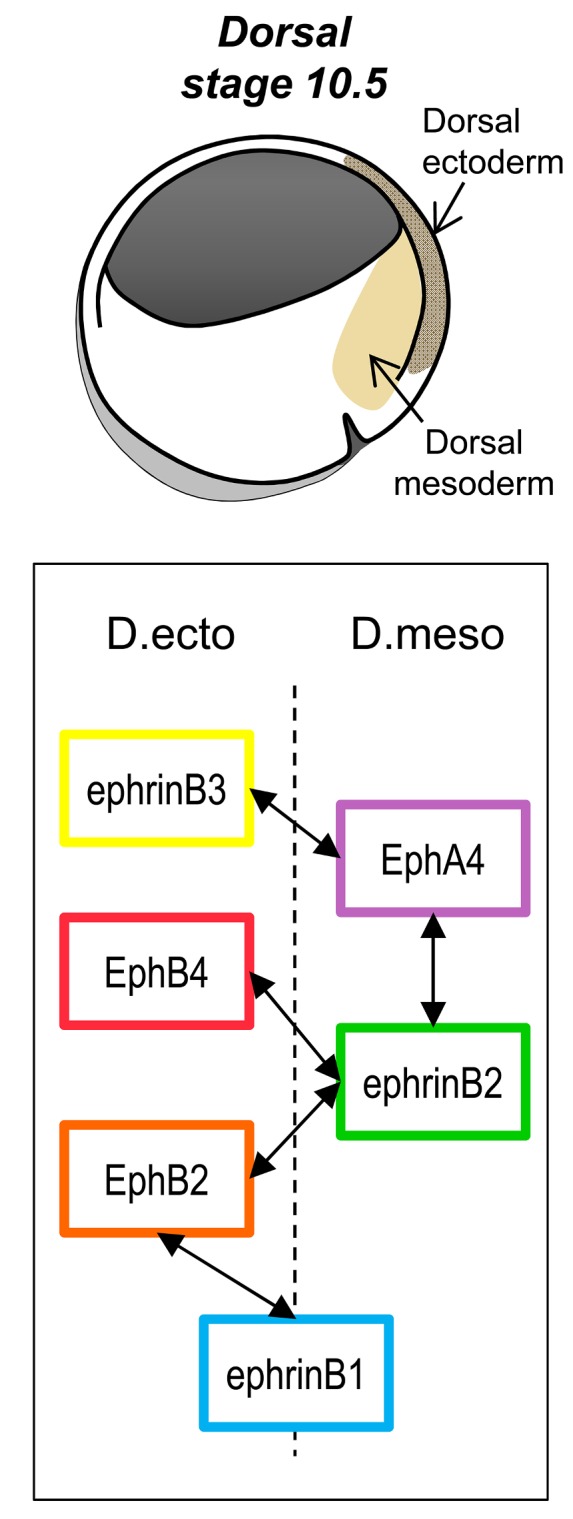
This study reveals how a system of multiple ephrin–Eph repulsive signals is used by embryonic cells to make the crucial decision to stay together or split into different tissues.

The authors examined one of the earliest separation events: that between the dorsal ectoderm (which gives rise to the nervous system and skin) and the mesoderm (which forms bone, muscle, and a variety of other tissues). They found that each tissue expressed its own set of ephrins and receptors: ephrin B3 selectively accumulated in the ectoderm, for instance, whereas receptor A4, to which ephrin B3 binds, is enriched in the mesoderm (Figure 1). Depletion of any one of the multiple ephrins or receptors, in either tissue, diminished the degree of separation of the two tissues, indicating the involvement of multiple signals in vivo. Switching the extracellular, but not intracellular, domains of different receptors switched their signaling abilities, showing that the specificity of the signal resides in the extracellular domain.

The tissue-specific expression of individual ephrin–receptor pairs was a critical aspect of boundary formation. When ephrin B3, normally expressed only in ectoderm, was expressed in mesoderm, it caused two portions of mesoderm to separate from each other, because the ectopic B3 interacted with the endogenous receptor A4. Similarly, when endogenous ephrin B3 interacted with ectopic receptor A4 in ectoderm, it caused separation of two portions of ectoderm. Remarkably, successful B3–A4 interaction did not depend on each being expressed in its usual tissue. When the endogenous expression of each was silenced, and then each was ectopically expressed in the opposite tissue, mesoderm and ectoderm separated normally.

Tissue separation is driven by the ephrin–receptor system but resisted by cell adhesion molecules called cadherins. The authors found that the strength of the two opposing forces determined the outcome: increasing the expression of cadherins reduced detachment, whereas decreasing their expression increased it. The mechanical act of detachment requires the contractile protein myosin, and chemical inhibition of myosin reduced tissue separation.

Finally, the authors found that a similar set of ephrin–receptor interactions explained separation of other tissues, including ectoderm from ventral mesoderm and the partitioning of the dorsal mesoderm to form the notochord. Although different in detail (different tissues express different sets and concentrations of ephrins and receptors), the same principles held: opposing tissues express specific ephrin–receptor pairs, whose interactions drive their detachment. Cells of a single tissue expressing a particular ephrin express little or none of the complementary binding partner, ensuring the integrity of the tissue. No single pair determines detachment by itself; the decision to separate is the sum of multiple ephrin–receptor interactions, as well as the strength of cadherins and the vigor of myosin contraction.

There is much more to learn here, including the consequences of the tissue-specific expression of individual ephrin–receptor pairs and how the level of each molecule is determined by the developmental eventspreceding its expression. The results presented in this study provide the framework for these future investigations.

**Rohani N, Parmeggiani A, Winklbauer R, Fagotto F (2014) Variable Combinations of Specific Ephrin Ligand/Eph Receptor Pairs Control Embryonic Tissue Separation.** doi:10.1371/journal.pbio.1001955

